# The reciprocal lagged effects of substance use and recidivism in a prisoner reentry context

**DOI:** 10.1186/s40352-017-0053-2

**Published:** 2017-06-07

**Authors:** Nathan Wong Link, Leah K. Hamilton

**Affiliations:** 1 Rutgers University, Camden, USA; 2 Temple University, Philadelphia, USA

**Keywords:** Substance use, Prisoners, Prisoner reintegration, Reentry, The life-course, Recidivism, Service needs

## Abstract

**Background:**

Much work has investigated the association between substance use, crime, and recidivism, yet little scholarship has examined these associations longitudinally among samples of recently released prisoners. We examine the lagged reciprocal effects of hard substance use and crime, among other covariates, in the context of the prisoner reentry process.

**Methods:**

We rely on data from the Serious and Violent Offender Reentry Initiative (SVORI) evaluation and employ cross-lagged panel models to examine short-term changes in substance use and crime over time among a large sample of high-risk, former prisoners (*N* = 1697).

**Results:**

Substance use marginally predicted increased odds of rearrest at one wave, and rearrest significantly (*p* < .05) predicted increased odds of substance use at another. As such, the results provide limited evidence for a degree of lagged mutual causation; associations vary over the reentry process and are complicated by other realities of life after prison. A key finding is that both behaviors are more consistently influenced by other factors, such as service needs and instrumental and emotional supports.

**Conclusions:**

Although there are relationships between drug use and criminal behavior, these behaviors alone are insufficient explanations for one another in an adult reentry population. Alternatively, the compounding social and personal needs of the reentry population, and the extent to which they received support or services to address these needs, appear to have the strongest influence on both behaviors in the reentry context.

How are drug use and crime linked, and can our understanding of this association inform health and social interventions among criminal justice populations? Across an array of health and social science disciplines, various theoretical perspectives posit differing patterns of causality in terms of the direction and/or escalation of the relationship between drugs and crime. In addition, some argue for external common causes of both drug use and criminal behavior. Importantly, most empirical research linking drugs and crime is cross-sectional, rather than longitudinal (Bennett & Holloway, 2009). Of the few studies that explore causal mechanisms, the focus is on initiation of drug use and criminal behavior among juveniles, as most drug use and deviant or criminogenic behavior begins in early adolescence (Belenko & Spohn, 2015). Notwithstanding the important contributions of these works, the discussion of causal pathways over time among adult populations has received far less attention, despite the possibility that findings could have critical implications for the health and well-being among inmates leaving prisons.

Approximately half of American inmates are symptomatic of substance abuse or dependence, almost all of whom will return to the community (Mumola & Karberg, 2006). Both relapse and re-offending are very common in the initial months post-release (Binswanger et al., 2012). In response to a recent call for cross-disciplinary work in public health and criminal justice (Gideon, 2013), we integrate the health, substance abuse, and criminological literatures by employing life-course and stress/strain paradigms and ask whether substance abuse affects later offending behavior, and vice versa, and simultaneously examine the other salient correlates that affect both behaviors among a sample of former prisoners. To this end, we rely on data from the Serious and Violent Offenders Reentry Initiative (SVORI), a panel study of the dynamics of “prisoner reentry”—the process whereby ex-offenders leave prison and re-enter (or “reintegrate” into) the community. Our findings indicate a complicated picture whereby use of hard drugs (heroin, cocaine, amphetamines) and crime (rearrest) do not consistently predict each other over time and—contrary to most theories of causation—crime better predicts later drug use than vice versa. Temporal relationships predicting drugs or crime varied throughout the reentry process, although some consistent relationships were found among other variables, such as the level of individual needs for services in reentry and instrumental and emotional family supports.

## Background

Theoretical explanations of the relationship between drugs and crime tend to fall into one of four categories: (1) drug use precedes deviant or criminal behavior, (2) deviant behavior or criminal behavior precedes drug use, (3) drug use and deviant or criminal behavior are mutually reinforcing or accelerating, and (4) drugs and crime or deviance are both the product of a common external cause. Some argue that different individuals’ drug and criminal behavior will fit into different categories at different times (Albery, McSweeney, & Hough, 2004).

One of the original heuristics explaining drugs and crime is Goldstein’s tripartite framework, although this framework is focused only on drugs and violent behavior. Goldstein (1985) argued that there are three rationales for the drug-violence relationship: people commit violent crimes because they are: (1) *psychopharmacologically* under the influence of drugs; (2) *economically compelled* to offend to fund drug use; and (3) *systemically* brought to crime by being involved in the violent environment of drug use and drug markets. Although this model is frequently cited as the logic behind the drug-crime nexus, it has had relatively little empirical testing (MacCoun, Reuter & Kilmer, 2003). Others have argued that it is limited in scope, and that the categories are too rigidly categorical and not mutually exclusive (Parker & Auerhahn, 1998). Some drugs, such as alcohol and to some extent cocaine, have been linked to increased violence (Bennett, Holloway, & Farrington, 2008), but many individuals who use these substances do not go on to commit crimes while under the influence (Belenko & Spohn, 2015). Furthermore, the economic-compulsive model has been shown to be a relatively rare phenomenon (White et al., 2009). Additionally, Goldstein’s (1985) model specifically addresses violent crime; however, many studies have demonstrated a link between property crime and drug use, and find weaker associations between drugs and violence than property offenses (Allen, 2005; Bennett, Holloway & Farrington, 2008; Parker & Auerhahn, 1998). Finally, Goldstein’s theory focuses heavily on the drugs-cause-crime explanation, which does not account for findings such as White et al. (2009) or Deitch, Koutsenok, and Ruiz (2000), both of which show that crime frequently precedes drug use. In short, although Goldstein’s (1985) model does appear to be supported in some circumstances, in other circumstances it is not.

Other theories tend to attribute both drugs and crime to some external cause. In some theories this is a singular factor such as the “general deviance syndrome” which purports that certain people have a relatively set propensity to commit deviant acts, of which both crime and drugs are common examples (Osgood, Johnston, O’Malley, & Bachman, 1988). Gottfredson and Hirshi’s (1990) concept of “low self-control” aligns with this construction of causation. Although there is some support for this theory and its applicability to the drug-crime correlation (Harrison, Erickson, Adlaf, & Freeman, 2001), scholars have noted that it does not appear to fully account for both behaviors at the same time (Osgood et al., 1988). Additionally, these theories tend to assume a constant level of low self-control or propensity for deviance across the life-course. Given this assumption it is not surprising that research on these theories is mostly focused on juveniles and young adults. However, as Belenko and Spohn (2015) note, individual drug-crime relationships vary over the life-course, which may also suggest that this perspective is again too limited.

A handful of scholars have identified a more developmental/life-course approach to both drugs and crime. This approach allows for the influence of multiple risk factors on both drug use and criminal behavior across developmental stages (Huizinga, Loeber, & Thornberry, 1993; White et al., 2009). Moore and Stuart (2005) take a bio-psycho-social perspective toward the relationship between drugs and violence that posits a multifactorial model whereby the risk of violence increases as a result of both distal (biological and developmental factors) and proximal (environmental) factors. Although the bio-psycho-social model is limited to aggression and tends to operate in the direction whereby substance abuse—along with other factors—leads to violence, this model aligns with central concepts in the criminological literature indicating the value of multifactorial models in explaining drug use and crime (Andrews & Bonta, 2010; Catalano & Hawkins, 1996).

Most developmental or life-course theories focus on childhood through young adulthood (Catalano & Hawkins, 1996; Moore & Stuart, 2005; White et al., 2009); however, Laub and Sampson’s (2003) age graded life-course theory offers a particularly useful theoretical framework for understanding the relationship between drugs and crime with an adult reentry population. They suggest that criminality and desistance is the product of both continuity and change across the life-course. As such, they employ the concepts of cumulative continuity (Moffitt, 1993) and state dependence (Nagin & Paternoster, 2000) to argue that a substantial degree of behavior is well established from early development, and that future behavior is the product of both adolescent development and cumulative life events. Specifically, major environmental stimuli—or “turning points”—can affect the trajectory of an individual life-course for the better or worse. Incarceration has been identified as a consequential, later-in-life turning point that might redirect the life-course (Laub & Sampson, 2003), although perhaps not always for the better (Cullen, Jonson, & Nagin, 2011). For example, Schnittker, Massoglia and Uggen (2012) found that incarceration was predictive of later psychiatric issues, which we know to be commonly comorbid with substance use and offending (Conway, Compton, Stinson, & Grant, 2006).

A parallel concept appears in the epidemiological literature, where it is argued that turning points are largely seen as life events that are part of “chains of social risk” or “exposure to causal factors” over the life-course (Kuh, Ben-Shlomo, Lynch, Hallqvist, & Power, 2003). Following this logic, returning to the community from prison and the various stressors that accompany attempting to reintegrate, such as seeking housing and employment, reestablishing family relationships and complying with conditions of release, are all opportunities for exposure to causal factors that could lead to re-initiating substance use or criminal behavior through a chain of social risks. Indeed, exposure to incarceration is often a negative experience (Schnittker, Massoglia & Uggen, 2012). Thus, this negative exposure opens up a potentially negative pathway where risk to reinitiate drug use or reoffend are more likely than it is for those who are not exposed to this negative experience.

Applying this framework to the drug-crime relationship, we can see that—among a population leaving prison—returning to drug use (or accelerating drug use if drug use took place while incarcerated) might be one event in the chain of cumulative events that leads to a pathway of re-offending. Conversely, re-offending might be part of a pathway that leads to a return to drug use. Further, the relationship between these two behaviors may be more indirect, whereby changes in something else—such as the presence of serious life stressors—after release are likely to affect the changes in drug use or offending, independently or as a part of cumulative pathway. Thus by examining the relationship between drug use and crime in a life-course paradigm we may better understand the nature of this relationship over time.

This framework also permits simultaneous examination of other important time-varying factors that may confound the association between substance use and crime after prison, such as omnipresent stressors and strain (Western, Braga, Davis, & Sirois, 2015), and a lack of services for practical needs in reentry, including but not limited to financial and legal assistance, employment/educational assistance, and healthcare assistance (Lattimore, Steffey, & Visher, 2010). Social stressors such as trouble finding work (Pager, 2008), dealing with large financial debt burdens (Harris, Evans, & Beckett, 2010; Roman & Link, 2015), family strife, and the stigma of ex-offender status (Uggen & Manza, 2005) can make the first few months out difficult and unstable. Mental health issues, whether present prior to, during, or after incarceration, may become exacerbated by the difficulties of the reentry process and lead to greater rates of reoffending than that experienced by those who do not have mental health issues (Bonta, Blais, & Wilson, 2014). Particularly in relation to violent crime, mental health conditions—especially those comorbid with substance abuse—appear to have an effect on offending (Link, Cullen, Agnew, & Link, 2016; Swanson et al., 1997).

According to some theoretical perspectives, these extensive stressors and gaps in service needs may push individuals to criminal behavior. Agnew’s (2006) general strain theory (GST) posits that negative stimuli, the removal of positive stimuli, or the failure to achieve a valued goal causes strain, which can foster deviant or criminal acts. Similarly, social epidemiologists and medical sociologists have examined life stressors and health and found that that stress from discrete life-events and chronic issues are linked to negative health outcomes, particularly mental health and substance abuse (Pearlin, Menaghan, Lieberman & Mullan, 1981). Stress in one area of life will often have spill-over effects on other areas of life (Thoits, 1995). For example, difficulty in finding work can result in financial strain on a whole family, resulting in marital difficulties. In addition to the ripple-effect of stressors, stress can accumulate over the life-course (Pearlin, Schieman, Fazio, & Meersman, 2005). Many major life stressors, particularly chronic issues and cumulative stressors, concentrate among people of color, young adults, and those of lower socio-economic status (Thoits, 2010). Thus, it is not difficult to understand that a typical group of reentering inmates will likely have high stress loads. Furthermore, this population often has poor coping mechanisms and little instrumental or emotional support, which are associated with an increased likelihood of health disorders (Taylor & Stanton, 2007).

Former prisoners face wide-ranging levels of stressors and service needs, and many lack adequate social supports and services. Fortunately, certain services are sometimes facilitated as a part of parole case management. Treatment programs for substance abuse and mental health conditions are some of the critical services which may help moderate both substance use behavior and criminal behavior over time. Research has suggested that need identification and service provision are critical elements of successful reentry and community corrections (Taxman, Young, & Byrne, 2004; Taxman & Belenko, 2012). Unfortunately, access to treatment services for offender populations is often difficult, with some studies showing that as much as 85% of inmates with substance abuse needs do not receive treatment (Mumola & Karberg, 2006). In the *Returning Home Studies*, La Vigne, Shollenberger and Debus (2009) found that parolees experienced difficulty accessing mental health treatment after release. Indeed mental health treatment is important for a host of reasons, but it is perhaps critically important vis-à-vis severing or moderating the linkages between the substance use and offending.

### Substance use and crime

Thirty-eight percent of parolees had a substance use disorder in 2012 (SAMHSA, 2014a). Over two-thirds of drug offenders will recidivate in the 3 years following release, and of parolees who recidivate, half of them were returned to prison for a technical violation—failed drug tests being a common reason for violations (Grattet, Petersilia, Lin, & Beckman, 2009; Langan & Levin, 2002).

Systematic review studies demonstrate clear associations between substance use and criminal behavior with varying degrees of strength (Bennett, Holloway & Farrington, 2008; Nurco, Hanlon & Kinlock, 1991). Although there does appear to be a higher rate of substance use and abuse in the subpopulation of individuals reentering the community than in the general population (SAMHSA, 2014b), this relationship has been demonstrated primarily through cross-sectional, correlative studies.

Existing studies examining this causal relationship have primarily focused on determining whether drugs or criminal behavior preceded the other. Among the studies of juveniles there is no clear consensus regarding the dominant causal pattern. Stenbacka and Stattin (2007) found higher rates of adult criminal activity among those who had used drugs during adolescence than among those who had not, regardless of other risk factors. Kandel et al. (2006) argue for a sequential pathway from alcohol and cigarettes to illicit drugs called the “gateway hypothesis”; however, this argument does not specify that drugs lead to other criminal behavior. Other studies such as White, Jackson, and Loeber (2009) and Menard, Mihalic, and Huizinga (2001) show delinquent behavior in juveniles tends to precede drug use. Furthermore, a systematic review of longitudinal studies found that there was an inconsistent relationship between substance use and delinquency (MacLeod et al., 2004).

Among adult populations there have been far fewer studies examining the causal relationship between drug use and criminal behavior. Nurco, Hanlon, Kinlock, and Duszynski (1988), in their study of addicted males, found that individuals who did not have a criminal history before their addiction emerged had a steeper escalation in their criminal behavior during their addiction periods than those who already had a criminal history before they became addicted. A smaller scale study by Allen (2005) demonstrated that some kinds of acquisitive crime appear to be correlated with heroin and crack use; however, qualitative interviews showed that for the majority of individuals, property crimes preceded their drug use. Bennett and Holloway (2009) interviewed a number of drug using offenders who identified a variety of rationales for their drug use, some of which indicated drug use preceding crime and some cases where crime followed drug use. Given the lack of statistical evidence on adult drug-crime causation, and the relatively mixed results from both the juvenile studies and limited studies of adults, more research is required to illuminate the relationship between drug use and offending in adults.

### The present study

The vast majority of studies that have examined the relationship between drug use and crime have done so using cross-sectional data (Bennett, Holloway, & Farrington, 2008) or retrospective qualitative accounts (Allen, 2005; Bennett & Holloway, 2009). The longitudinal studies that examine this relationship have focused on adolescents and young adults, and frequently are concerned with initial onset of drug use and criminal behavior. We argue that understanding this relationship in adults, particularly among inmates who are reentering the less-controlled environment of the community, is a critical and understudied part of the drug-crime nexus. Simultaneously, it could also be of potentially great policy relevance given that the U.S. is perhaps in the process of addressing its addiction to incarceration, and moving toward an orientation that places greater emphasis on public health versus criminal justice vis-a-vis substance use and abuse.

The SVORI dataset provides the opportunity to examine a large sample of reentering ex-offenders over the span of 17 months from baseline through three post-release time points. Using path analysis we can illuminate the relationships between these two behaviors over time. Although we cannot completely control for the potential for spuriousness, these panel data and this type of model comes closer to a causal understanding of the drug-crime nexus than previous analyses by establishing temporality, change over time, and controlling for major covariates—including critical factors such as past levels of our outcome measures.

## Data and methods

We take advantage of a subset of male inmates from the Serious and Violent Offender Reentry Initiative (SVORI) dataset (Lattimore et al., 2012; Lattimore & Visher, 2013) to interrogate whether rearrest and substance use have impacts on each other after release from correctional institutions. The subpopulation focused on the 1697 adult male offenders across four data collection waves: 30 days prior to release, and 3-, 9-, and 15-months post-release. The SVORI dataset is the largest post-release from prison study to date. At 3 months, 58% (984) were successfully re-interviewed; 61% (1035) were interviewed at 9 months; and 66% (1113) at 15 months. Forty-two percent of respondents were successfully interview at each wave.

The SVORI project was originally conducted by a collaborative research team from RTI International and the Urban Institute (Lattimore et al., 2012). The study used a propensity score matched or better research design (2 sites used randomization processes) to test reentry interventions focusing on service provision after release from incarceration. This evaluation was conducted in Ohio, Indiana, Iowa, Kansas, Maine, Maryland, Missouri, Nevada, Oklahoma, Pennsylvania, South Carolina and Washington. For this study the treatment and comparison groups are analyzed together as our particular interest is not to evaluate the intervention, but rather to examine the patterns of crime and illicit substance use behavior over time. The participants in the study had histories of serious and violent criminal behavior as defined by the evaluation sites, with self-reported high levels of substance use and abuse (Lattimore et al., 2012).

### Endogenous variables

The first endogenous measure examined arrest for any crime following release from prison. This variable is measured as official recidivism using a dichotomous (1 = Yes, 0 = No) response for commission of any criminal offense. Official arrest recidivism data are derived from the FBI’s National Crime Information Center (NCIC). These data captured arrests occurring either within or outside of a respondent’s home state. This outcome is measured at each wave of post-release data collection – 3, 9, and 15 months post-release. Official rather than self-report crime data were used as the self-report data suffer from substantially more missing data due to subject attrition (NCIC data were available for 93% of the sample—1581 respondents). For those 7% missing NCIC data, reincarceration data were used as a proxy for rearrest.^1^ Any arrest for a new criminal offense was used since there are theoretical hypotheses which posit a crime-to-fund-drugs rationale (more likely to be property offenses) as well as drugs are part of a violent subculture or drug use psychopharmacologically causes criminal—and sometimes violent—behavior (Speckart & Anglin, 1985; Goldstein, 1985). We recognize that although rearrest does not account for all crime committed, and may include cases where individuals were erroneously arrested, it is a more reliable measure than the self report data available for this sample and rearrest is a primary concern for scholars of the reentry process, as it is what truly can change whether someone reintegrates into a community or is again removed to prison or jail.

The second endogenous measure examined substance use, measured as use of cocaine, heroin, or amphetamines in the last 30 days prior to each interview including baseline pre-release interview. This variable was coded as a dichotomous (1 = Yes, 0 = No) outcome. We selected this measure of substance use as previous research has found that heroin, cocaine, and crack, and amphetamines generally show the strongest associations with criminal actions (Bennett et al., 2008; Boles & Miotto, 2003).^2^

In this particular analysis, the above-described variable substance use is used as the predictor in the analysis of determining the likelihood of rearrest. Simultaneously, rearrest is used as a predictor of the likelihood of substance use. Additionally, earlier wave measurements of both substance use and arrest are used to predict later waves of substance use and re-arrest. As such, our empirical test is strict as paths from substance use to later rearrest and vice versa do not represent the impact of one on the other, but one on recent changes in the other.

### Exogenous variables

In addition to the central variables of drug use and crime, we have incorporated a number of covariates in the model that are frequently associated with drug use and crime. Included in the analysis were two variables that provided an understanding of the subjects’ criminal histories. First, a continuous variable indicating the number of previous arrests before baseline data collection was included in the model. Second, given that the association between crime and substance use is stronger for property offenders (Bennett, Holloway, & Farrington, 2008), a variable indicating the type of offense for which the subject was incarcerated most recently was included. This variable was measured with property offense as the indicator category and other types of offense as the reference category. Since mental health status is related to both criminal activity and drug use, we inserted a time-varying dichotomous indicator of mental health status using the item: “Do you currently need mental health treatment?” Similar to studies controlling for opportunity by including “time on the street” in the analysis, reincarcerated status was controlled with dummy indicators to identify respondents who, according to official records, were incarcerated at each follow-up interview point and were therefore unable to be rearrested or relapse in the community.^3^ Supervision status measured whether *s*ubjects were on probation or parole (Yes = 1, No = 0) at each wave.

A continuous variable, service needs, was used to measure the degree of need for practical reentry services. This variable—measured at each wave—was created with 28 items (Yes = 1, No = 0) probing five different domains of reentry needs: employment needs, health services needs, family needs, child services needs, and transitional service needs (Lattimore, Steffey & Visher, 2010). Scores for each individual were generated by summing the number of reported needs and dividing by the number of items (28 items for those with kids, 23 for those without). Thus, scores ranged from 0 to 1. These scores were then multiplied by 100 so that the combined service needs scale had a potential range from 0 to 100. Cronbach’s alpha was .70, .74, .75, and .75 for baseline, 3-, 9-, and 15-months, respectively.

Finally, success in avoiding both relapse in substance use and re-offending is improved by having strong family support (Spohr, Suzuki, Marshall, Taxman, & Walters, 2016; Taylor, 2016; Thoits, 2010) and receiving mental health and drug and alcohol treatment. Family support was measured as an index variable addressing issues such as level of family problems and whether family could be confided in. The scale ranged from 0 to 30 with higher values indicating higher support. Cronbach’s alpha was consistently .87 at 3-, 9-, and 15-months. Mental health and substance abuse services were measured as time-varying dichotomous indicators measuring whether the respondent received treatment since release from prison or the last interview.

Most demographics were measured at baseline and were time invariant for the analysis. A dichotomous variable for race with “African American” as the indicator category and all other racial categories as the reference category was included. Age was measured in years at baseline. Education was measured as a dichotomous variable with completion of at least high school education or GED as the indicator variable. Having children at the time of the baseline interview was included in the analysis with having children as the indicator category and no children as the reference category. Marital status or having a serious partner was included as a time-varying dummy indicator. Finally, a measure of employment was identified at each wave of post-release data collection. Respondents were assigned a “1” if they reported having a legitimate job since the last interview, and a “0” if they did not have legitimate employment. To control for variation in services received by subjects as a result of the SVORI intervention we included a dichotomous indicator of whether the subjects were in the treatment condition of the SVORI intervention.

### Analytic strategy

We analyzed the associations between substance use and arrest within a cross-lagged framework. The panel nature (repeated observations of the same people over time) of these data is leveraged here for two related reasons. First, our key variables are time-varying, thus allowing us to see how they change in the prisoner reentry process. More importantly, analyzing the data longitudinally allows for previous levels of the same variables to be controlled. Therefore, paths from one construct (X) to another (Y) no longer reflect the impact of X on Y, but X on *short-term changes* in Y. In this scheme it is still impossible to preclude the possibility that some third, time-varying variable Z is causing both the changes in X and Y. However, this method is considered to be a marked improvement over traditional cross-sectional models that are under threat of endogeneity and time-order problems (Wooldridge, 2010), which is critical for analyzing variables such as substance use and crime. Indeed, analyzing substance use and crime within a cross-sectional framework would likely suffer from artificially inflated coefficients due to simultaneous causation. For this reason, we are exclusively examining the lagged effects of crime and substance use on one another.

**Fig. 1:**
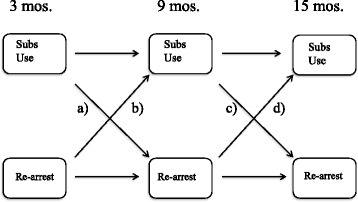
Conceptual model

Substance use and recidivism are treated as endogenous—they affect each other over the course of reentry. The core model indicating the hypothesized paths for the two key variables (with covariates omitted) is shown in Fig. 1. Pathway *a* shows the effect of substance use from release until the 3-month interview on arrest between 3 and 9 months. Pathways *a* and *c* test the drugs-cause crime model by which substance use from one time period impacts changes in arrest later in time. Conversely, pathways *b* and *d* test the crime-causes-drugs model, whereby getting arrested affects changes in substance use since the previous interview.

Because the key variables of interest are dichotomous (substance use and rearrest), generalized structural equation modeling (GSEM) in Stata 13 was used to estimate this path model. Substance use and other covariates were regressed on re-arrest and rearrest and other covariates were regressed on substance use.

Missing data resulting from subject attrition is addressed here using two different approaches. First, the GSEM’s Heckman probit correction (−heckprobit-) (StataCorps, 2013) was used to address sample selection bias due to attrition at follow-up waves (3-, 9-, and 15-months). Unlike the two-step Heckman correction that models the selection equation using probit regression, obtains the inverse mills ratio (IMR) for each case, and includes the IMR in an OLS model, the Heckman probit correction in GSEM uses latent variables and probit regression only (StataCorp, 2013). In the GSEM approach, a variable indicating whether the respondent selected (SELECTED) into the sample at that interview was used. A latent variable (L) with a variance constrained to 1 affects the outcome of interest (e.g., SUBS USE ← L), in addition to affecting the selected variable (SELECTED ← L), with the latter path’s coefficient constrained to 1. Paths from independent variables are drawn toward both the outcome of interest and the selected variable. Age was removed from the primary equations for two reasons. First, it showed collinearity problems with the key family support variable, and two, it did not significantly predict our recidivism outcomes, perhaps because the nature of the sample is older than most offender samples. We did, however, include age in equations predicting selection. Otherwise variance inflation factors indicated no issues of multicollinearity in the model. The second approach employed for handling addressing missing data is through GSEM’s maximum likelihood estimation procedures in Stata 13. This approach uses equation-wise deletion rather than listwise deletion, which does not automatically drop cases that have some missing data. Rather, similar to pairwise present analysis, it uses all of the data available to it when estimating specific model parameters (StataCorp, 2013).

## Results

Table 1 provides the descriptive statistics for the key variables. Substance use outcomes varied over time, with the highest percentage of respondents reporting substance use during the month before entering prison (35%). Upon release, 8% reported use by the 3-month interview, and 13% and 11% by the 9-month and 15-month interviews, respectively. Arrest prevalence increased sharply between 3- and 9-months, and remained fairly stable between 9- and 15-months.

**Table 1:** Summary statistics

	*N*	*M* or %	*SD*	Range
Dependent Variables				
Substance Use				
Baseline substance use	1694	35%	48%	0–1
T2 substance use	984	8%	27%	0–1
T3 substance use	985	13%	34%	0–1
T4 substance use	921	11%	31%	0–1
Rearrest (1 = Yes, 0 = No)				
T2 rearrest	1697	16%	48%	0–1
T3 rearrest	1697	32%	46%	0–1
T4 rearrest	1697	30%	46%	0–1
Time-varying Covariates				
General Social and Personal Needs Scale				
T2 needs	984	42.70	20.68	0–100
T3 needs	1033	43.29	21.46	0–100
T4 needs	1113	44.60	22.20	0–100
Re-incarcerated (1 = Yes, 0 = No)				
T2 reincarcerated	984	7%	26%	0–1
T3 reincarcerated	1035	26%	44%	0–1
T4 reincarcerated	1113	35%	48%	0–1
On Supervision (1 = Yes, 0 = No)				
T2 supervised	982	83%	37%	0–1
T3 supervised	1032	68%	47%	0–1
T4 supervised	1108	53%	50%	0–1
Family Emotional Support Scale				
T2 family support	959	22.36	4.89	0–30
T3 family support	955	21.56	4.96	0–30
T4 family support	901	21.59	4.88	0–30
Employment (1 = Yes, 0 = No)				
T2 employment	983	62%	49%	0–1
T3 employment	983	69%	46%	0–1
T4 employment	922	66%	47%	0–1
Married/Partner (1 = Yes, 0 = No)				
T2 married/partner	983	57%	49%	0–1
T3 married/partner	1035	64%	48%	0–1
T4 married/partner	1113	57%	50%	0–1
Mental Health Issue (1 = Yes, 0 = No)				
T2 mental health	982	20%	40%	0–1
T3 mental health	1032	21%	41%	0–1
T4 mental health	1110	25%	43%	0–1
Received MH services (1 = Yes, 0 = No)				
T2 MH services	973	8%	27%	0–1
T3 MH services	974	8%	27%	0–1
T4 MH services	910	8%	27%	0–1
Received AOD services (1 = Yes, 0 = No)				
T2 AOD services	984	26%	44%	0–1
T3 AOD services	984	23%	42%	0–1
T4 AOD services	917	19%	39%	0–1
Time Invariant Covariates				
Age at baseline	1697	29.20	7.29	18–73
African American (1 = Yes, 0 = No)	1697	57%	50%	0–1
SVORI participation (1 = Yes, 0 = No)	1697	51%	50%	0–1
Index offense-property (1 = Yes, 0 = No)	1697	19%	39%	0–1
Prior arrests	1697	13.57	20.27	0–300
High school education	1697	59%	49%	0–1

Means and standard deviations for dichotomous variables reported as percentages

Odds ratios for arrest and substance use from the multivariate structural equation model are shown in Tables 2 and 3. The key theoretical pathways of interest here are how substance use and arrest influence each other over time. Testing the more common drugs-cause-crime theoretical pathway, substance use reported by the 3-month interview did not have significant lagged impacts on arrest outcomes at the 9-month interview. However, a marginal effect appeared by the 15-month interview: using heroin, cocaine, or amphetamines at the 9 month interview increased the odds of changes in arrest at the following interview by 72% (*p* < .10), net of other covariates.

**Table 2:** Re-arrest Outcomes (ORs) estimated via GSEM, *N* = 1696

Re-arrest	T2 (3 months)		T3 (9 months)		T4 (15 months)	
	OR	*SE*	OR	*SE*	OR	*SE*
Prior Re-arrest			2.320**	.660	3.462***	.777
Prior Substance Use			.959	.354	1.715†	.548
#Prior Arrests	.999	.006	1.003	.004	.999	.005
Employed	.389***	.093	.827	.186	.759	.168
Needs	.995	.007	1.013*	.006	1.017**	.006
Mental Health	1.252	.408	.951	.265	.824	.228
MH Services	1.470	.179	.993	.382	1.140	.451
AOD Services	.765	.217	1.441	.341	1.087	.290
Married/Partner	.779	.179	.766	.163	1.079	.234
African American	1.886*	.490	1.318	.294	1.289	.281
SVORI participant	.963	.218	.800	.148	1.027	.209
H.S. Education	.433***	.101	.670†	.142	.885	.188
On Supervision	.808	.224	.634*	.136	.934	.196
Family Emotional Support	.982	.024	.982	.021	.972	.022
Property Offense	.748	.239	1.431	.361	1.628†	.546
Reincarcerated	29.644***	10.473	6.780***	1.630	5.543***	1.373
Model Log-likelihood	*df*	AIC	BIC			
-5744.573	123	11,735	12,403			

†*p* < .10, one-tailed

*p < .05, **p < .01, ***p < .001, two-tailed

**Table 3:** Substance Use Outcomes (ORs) estimated via GSEM, *N* = 1696

Substance Use	T2 (3 months)		T3 (9 months)		T4 (15 months)	
	OR	*SE*	OR	*SE*	OR	*SE*
Prior Substance Use	2.710***	.435	6.582***	1.619	5.467***	1.123
Prior Re-arrest			1.672*	.357	.965	.198
Employed	.809	.123	.773	.129	.954	.183
Needs	.998	.004	1.019***	.005	1.007	.005
Mental Health	1.744**	.343	1.213	.247	1.316	.278
MH Services	.855	.236	.333**	.131	.859	.262
AOD Services	1.258	.205	1.029	.186	1.411	.279
Married/Partner	1.014	.153	.598**	.357	.924	.168
African American	.863	.142	.924	.162	.768	.144
SVORI participant	1.152	.172	1.069	.171	1.195	.209
H.S. Education	1.103	.178	1.174	.200	.976	.178
On Supervision	.602**	.102	.846	.139	1.123	.199
Family Emotional Support	.956**	.014	.984	.015	.977*	.019
Property Offense	1.100	.197	1.332*	.257	1.177	.235
Reincarcerated	2.847***	.615	2.049***	.369	2.827***	.525
Model Log-likelihood	*df*	AIC	BIC			
-5744.573	123	11,735	12,403			

†*p* < .10, one-tailed

*p < .05, **p < .01, ***p < .001, two-tailed

Regarding the crime-causes-drugs theoretical pathway, results show arrest at one time period significantly increases the likelihood of using hard substances at the next interview. Being arrested by the 3-month interview increased the odds that the person reported using heroin, cocaine, or amphetamines between the 3- and 9-month interviews by 67% (*p* < .05).

Other significant results were found that accord with prior literature. Importantly, higher needs are significantly associated with a higher likelihood of *both* rearrest and substance use (significant at 9 months for substance abuse; at 9 and 15 months for rearrest). Note that although the impacts of needs on these outcomes appear small (i.e., small ORs), they are in fact rather large given the measure is a 100-point scale, while many other variables were dichotomized or measured using smaller scales. In addition, emotional family support was associated with reductions in the likelihood of reporting substance use at 3 and 15 months (*p* < .01 and *p* <. 01, respectively). Being under criminal justice supervision (i.e., probation or parole) was associated with a decreased likelihood of reporting substance abuse by the 3 month interview (*p* < .01) and rearrest by the 9 month interview (*p* < .05). Employment was linked with lower likelihoods of recidivism, yet being employed showed no significant impacts on substance use. Mental health and mental health service variables were not significantly associated with any rearrest outcomes. However, having a mental health issue did increase the likelihood of reporting hard drug use, and was significant at the nine-month interview (OR = 1.74, *p* < .01). Receiving mental health treatment was generally associated with a lower likelihood of using substances, and significantly reduced the odds of substance use by 66% (*p* < .01) at the three-month interview. Surprisingly, receipt of substance use treatment services did not return significant results with regards to later substance use or criminal behavior.^4^

As expected, the stability coefficients that link earlier outcomes with later outcomes (i.e., earlier substance use predicting later substance use) indicated that both substance use and arrest predicted themselves very strongly over time. Interestingly, the impacts on themselves became stronger over time, which—from a life-course perspective—has both theoretical and policy implications.

## Discussion

Substance use and crime often go together, yet why this is so remains unclear. Over the course of 30 years, two types of research have informed the discussion on how drugs and crime relate to one another. The first type leverages longitudinal studies and has focused on the phenomenon of the onset of substance use and criminality—specifically, which behavior occurred first among juveniles, and did it lead to the other behavior? This body of research is hugely important for theory because it delineates a clear temporal ordering between the two in the context of adolescent development, and in doing so is able to obviate the perennial issue of reverse causality. Concurrently, the second critical and large body of research has examined the strong and persistent cross-sectional associations between substance abuse and crime among adults (Bennett & Holloway, 2009), often using samples of prisoners. From this literature we have gained knowledge on which types of offenders use which types of substances, how frequently, and how strongly each behavior is connected to the other.

Notwithstanding the importance of these two discrete types of research (longitudinal samples of adolescents/young adults and cross-sectional samples of adults), a dearth of literature remains for longitudinal studies of justice-involved adults, particularly with respect to adults in a reentry population. The current work sought to capitalize on the strengths of both types of research in this area by longitudinally examining drugs and crime among a high-risk, adult, former prisoner population. Using panel data we were able to identify the lagged effects of each on the other over time, thus reducing the problem of endogeneity bias (a strength of the samples examining onset). These results provide insights into reducing re-offending, substance abuse and other social hazards characteristic of prisoner reintegration (a strength of the adult population research).

Although both drug use and rearrest were strongly self-reinforcing and accelerating over time, providing credence for the theoretical argument that behavioral pathways for crime or drug use can become deeply ingrained, they were not necessarily part of the “causal chains of risk” for one another. Results showed only one marginally significant effect of drugs➔crime and one significant result where crime➔drugs. Thus, we found very limited support for a reciprocal relationship between substance abuse and rearrest over time in the reentry process.

Though very little work has examined the *lagged* impacts of drug use on crime, life-course paradigms suggest that substance use may either facilitate continuity or redirect lives in untoward directions, such as criminal activity. Despite the life-course approach and the commonly held belief that substance abuse leads to crime, in our models drug use only marginally predicted later arrest at one wave. This means that—at least vis-à-vis arrest—we found little support for the idea that those who use drugs are necessarily on a “bad course”—an important finding for both correctional authorities tasked with classifying and predicting people as likely recidivists, and for the classified-as-likely-recidivists themselves. The reverse pathway—that crime can significantly increase later substance use—is less well understood. Respondents who were arrested in one wave were significantly more likely to report increases in substance use by the following wave. One possible explanation for this relationship can be drawn from theoretical frameworks in the health disparities literature which suggest that stress, strain, and stressful life events, especially when coping tools and social support are absent, can have deleterious affects on health and well-being (Pearlin et al., 2005; Thoits, 2010). Applied to the present finding, if being arrested shortly after release from prison qualifies as a stressor or stressful life event, it follows that substance use and abuse might occur as a result. The stress associated with this type of justice involvement following release from prison brings a host of anxieties, the most prominent of which may be the possibility of returning to incarceration. With little resources, social support, and other coping mechanisms, it is not far-fetched that, following arrest, returning to drugs is a common reality for many. Alternatively, literature on anti-social peers or networks formed during incarceration could provide an explanation for later substance use behavior. Future research may wish to consider ethnographic studies of this time period to better identify what conditions lead to substance use in a post-release population. Noting that there are instances where drugs and arrest appear to influence each other among this reentry sample, the results make it clear that both are more often shaped by a dynamic web of social and personal needs that can redirect their lives toward or away from salubrious outcomes. These factors broadly can be sorted into needs-based factors and support factors.

The scale of service needs in our models not only predicted rearrest but also significantly predicted substance use. Indeed, of all the covariates in our models (time-varying and time-invariant), the service needs scale predicted increases in *both* types of types of behavior more consistently than any other. The finding that the SVORI participants had a high degree of service needs upon release to the community is not surprising, yet the fact that these scores consistently and strongly predicted both outcomes underlines the salient role that other structural, non-behavioral factors play in influencing both problem behaviors.

There is much evidence that stressors and strain precipitate health problems, including substance abuse (Dohrenwend & Dohrenwend, 1974; Thoits, 1995). Unsurprisingly, given the frequency of comorbidity of mental illness and substance use (Hills, 2000), mental health needs of the former prisoner increased the likelihood of substance abuse in this analysis, independently of the general scale of needs. Reentry populations face many stressors, including nearly insurmountable hurdles and hardship once released (Petersilia, 2003; Travis, 2005; Western et al., 2015), high rates of mental health problems (Schnittker, Massoglia, & Uggen, 2012), and returning to areas of neighborhood disadvantage (Boardman et al., 2001). These needs are likely a perennial source of stress and frustration. Thus, the multistep chain could be that stress and strain mediates the associations seen between needs and increased substance use (i.e., needs➔stress➔substance use).

From a criminological perspective, that higher needs increased recidivism in reentry can also be viewed through a general strain lens (GST; Agnew, 2006). Released inmates face substantial adversity, including difficulty securing employment (Pager, 2008), discrimination and stigma (Uggen & Manza, 2005), criminogenic and jobless neighborhoods (Clear, 2007), and hefty financial debt burdens (Harris et al., 2010; Roman & Link, 2015; Visher et al., 2004). GST—as a corollary theory to the medical sociological theory of stress on health—predicts that stress and strain such as these will increase criminality, especially if such criminality is seen as addressing the discomforting effects of the experienced strain. Since health data show that stressors tend to concentrate among low-SES and people of color (Thoits, 2010), it follows that stressors among these populations may lead to a greater risk of reoffending. If this is the case, then the causal chain with respect to recidivism among this sample would be needs➔stress/strain➔recidivism.

Research on reentry populations has to some extent acknowledged the difficult and changing environment of the transition to the post-release environment. Taxman, Young, and Byrne’s (2004) model for reentry argues for three phases of the reentry process: *institutional*, *structured*, and a *community reintegration*. This model suggests that there are changing circumstances during reentry, which warrant different services over these stages of reentry. The results of this study appear to confirm that the needs of returning inmates appear to change over time, and that drug use and rearrest may at times be one of a compendium of other needs that influence later drug and criminal behavior. These results suggest that studies centering on assessing and identifying a multitude of needs for services in reentry and repeating this process over the course of the reentry period would be more appropriate than a narrow focus on the drug-crime connection in the reintegration process.

This is further corroborated by the results indicating that support systems—whether instrumental or emotional—can interrupt these pathways from needs to crime or substance use. Being under supervision decreased the likelihood of returning to substance use as well as reoffending, although this result was not consistent over time. Thus, probation/parole supervision might protect against the consequences of a stressful transition from incarceration to community. Regarding emotional family support, while we found no evidence that it significantly decreases rearrest, we did find that increases in emotional family support decreased substance use at two of the three follow-up waves. This finding is in accord with theoretical frameworks arguing that familial support can temper the ill effects of stress and strain on health hazards (Taylor & Stanton, 2007; Thoits, 1995). Receiving mental health services also appears to decrease the return to substance use, although treatment of substance abuse problems did not have a significant impact on later drug use. The significant result of mental health treatment may indicate that individuals with mental health conditions use illicit substances to self-medicate, and in treating the underlying condition the desire to use is lessened. The lack of results with regard to substance abuse treatment may reflect the broad range in the quality of treatment provided to individuals in the criminal justice system across agencies, counties, and states (Taxman, Cropsey, Young, & Wexler, 2007).

Our findings are qualified by a set of limitations. First, as in all reentry studies and most panel studies, there is a non-trivial amount of missing data due to subject attrition in the SVORI data, which could introduce bias into our models. Other than implementing Heckman probit models to address this issue statistically, we performed standard checks to identify patterns of missingness and, similar to other recent analyses of the SVORI data (Link & Roman, 2017; Stansfield, Mowen, O’Connor, & Boman, 2016) did not find significant differences between attriters and nonattriters on key variables in our analysis. Nonetheless, the potential threat to validity introduced by attrition remains an issue. Second, since our measure for drug use derived from self-report methods, it is possible that this variable is subject to bias due to underreporting and social desirability bias (see, however, Brown, Kranzler, & Del Boca, 1992). And third, the SVORI data are limited to the largely medium- and high-risk male prison population from 12 states who were recruited for the original SVORI project through convenience sampling of the target sub-population. Therefore, the findings may not be perfectly representative of the target population and not generalizable to the entire population of prisoners in the United States.

## Implications and conclusions

On a practical level, our findings suggest that—in addition to addressing known criminogenic areas such as substance use in the reintegration process—community correctional agencies such as probation and parole should seek to identify the varied needs of their returning inmate populations, and repeat this process over the course of parole in order to provide appropriate services for multiple compounding needs. This seems especially important given the recent decline in incarcerated populations (Frazier, Sung, Gideon, & Alfaro, 2015). Like Taxman and Belenko (2012)—who argue that the public health and public safety goals of community corrections need not be in conflict with one another—our data suggest that community correctional agencies may better achieve the goal of reducing both drug use and reoffending by adopting a social and health service paradigm over a strictly law enforcement model. The influence of service needs on reentry is an encouraging finding since they are dynamic and amenable to intervention. One way in which these needs could be addressed would be to place emphasis in prisoner reintegration on models or programs whereby services for these salient reentry needs are identified during incarceration and facilitated upon release (Gideon, 2013; Hamilton & Belenko, 2016), analogous to public health models that “reach in” to institutional corrections and begin to identify and link prisoners with appropriate community health resources (Conklin, Lincoln & Flanigan, 1998). Should this be achieved, the benefits could span from improved health to reduced recidivism.

These results seem to suggest that substance use is perhaps not as strong a predictor of reoffending as many common risk assessments assume (e.g. LSI-R, Andrews & Bonta, 2010; HRC-20, Douglas & Webster, 1999). While a substantial body of literature argues that substance abuse is a significant predictor of reoffending (e.g. Huebner & Cobbina, 2007; Stenbacka & Stattin, 2007; Wilson, Draine, Hadley, Metraux, & Evans, 2011), other work questions the causal link between substance abuse and crime, particularly violent crime (Parker & Auerhahn, 1998; Gelles & Cavanaugh, 2005). While it is premature to suggest that these risk models are misidentified, further examination of the longitudinal relationships between drug use and crime among former prisoners is needed. Particularly, studies of reoffending outcomes in light of the interactions between substance use behavior and the constellation of other needs that affect parolees may help provide a more accurate portrait of the role of substance use as a predictor of recidivism.

Contingent on further replication of these results, some changes to policy could be implied. Currently, much attention within supervision agencies is put on identifying high-risk candidates likely to re-offend. A primary mechanism used to identify these candidates is mass drug testing. To be sure, drug testing should be a component in supervision, but by shifting the use of drug test results toward an effort to identify and address major service needs, rather than focusing primarily on risk identification, public safety and public health might be improved simultaneously.

The results of this study also suggest that although temporal relationships between drug use and rearrest may occur, these associations alone are insufficient explanations for both behaviors in an adult reentry population. Rather, the compounding social and personal needs of the reentry population, and the extent to which they received support or services to address these needs, appear to have the strongest influence on both drug use and rearrest in the 16 months after release from prison. As such, future research and policy may find better success in reducing both re-offending and substance use by addressing former inmates’ post-release life context including identifying service needs, facilitating social supports, and providing opportunities for high quality treatment.

## Footnotes

1 We recognize the pros and cons of this strategy. While respondents who were reincarcerated were almost certainly rearrested, there could exist respondents who were rearrested but not reincarcerated. Still, we expect that this approach is superior to dropping these cases altogether.

2 This analysis was also run with a variable for those cocaine and heroin only, as these drugs are most commonly associated with crime (Bennett et al. 2008). Results showed similar results for associations between arrest and drug use.

3 Due to concerns that this variable might be problematic by artificially eating up variation in the outcomes, models were also run with this variable omitted. Results were nearly identical with this control removed.

4 While we do not wish to overstate a null effect, it should be noted that although the study measures receipt of substance use treatment, the quality of this treatment cannot be ascertained from the data. Evidence suggests that that treatment service availability and quality can differ significantly between states and between institutions (Taxman, Perdoni & Harrison, 2007). Though the sample sizes would become rather small, future work might consider in-depth, intrastate analyses of the link between substance use and offending.
